# Engineered immune cell therapies for solid tumors: pharmacological advances, clinical outcomes, and future directions

**DOI:** 10.3389/fphar.2025.1614325

**Published:** 2025-06-12

**Authors:** Ziyad M. Althafar

**Affiliations:** Department of Medical Laboratories Sciences, College of Applied Medical Sciences in Alquwayiyah, Shaqra University, Riyadh, Saudi Arabia

**Keywords:** solid tumor immunotherapy, engineered immune cell therapy, CAR-T cell therapy, tumor-infiltrating lymphocytes, pharmacological mechanisms

## Abstract

Solid tumors, accounting for around 90% of human cancers, present unique challenges due to antigen heterogeneity, immunosuppressive microenvironments, and limited accessibility for conventional pharmacotherapies. Immunotherapies, particularly engineered immune cell therapies, exploit the immune-tumor interplay, offering novel pharmacological strategies for solid malignancies. Genetic engineering enhances adoptively transferred cells, such as T cell receptor therapy, chimeric antigen receptor (CAR)-T cells, tumor-infiltrating lymphocytes (TILs), natural killer cells, and CAR-macrophages, by optimizing their targeting and effector functions. Clinically, TIL delivery has shown significant responses in advanced melanoma, with lifileucel gaining United States FDA approval as a pioneering TIL therapy for solid tumors. Ongoing trials further explore these approaches, revealing promising outcomes in overcoming immunosuppressive barriers. However, challenges persist, including optimizing combination therapies, streamlining manufacturing for off-the-shelf accessibility, and mitigating pharmacotoxicity. This review synthesizes recent advances in engineered immune cell therapies for solid tumors, emphasizing their pharmacological mechanisms, clinical efficacy, and translational potential. By addressing current hurdles, such as enhancing tumor penetration and minimizing adverse effects, this article outlines future directions to refine these therapies as safe, effective pharmacological tools in oncology.

## 1 Introduction

Cancer-associated mortality rates are increasing globally every year (Maalej et al., 2023). In the United States, 2,001,140 new cancer cases and 611,720 cancer deaths were reported in 2024 alone. Conventional cancer therapies including chemotherapy, radiation therapy, and surgery, possess many drawbacks and numerous recurrent and metastatic cancer patients still through dismal outcomes (Albano et al., 2021). On the other hand, various systemic therapies such as immune checkpoint inhibitors, targeted therapies, and chemotherapy are used to treat metastatic solid tumors. However, most of the patients with metastatic solid tumors are treated with available and incompetent conventional therapies, thus requiring additional therapeutic options. The occurrence of solid cancers or solid tumors is very high, where it is estimated that around 80% of all types of tumors originate from a subset of solid organs such as the ovary, colon, lung, prostate, and breast (Najafi et al., 2021).

Solid tumors are derived mostly from epithelial tissue and play a major role in mortality and morbidity worldwide, wherein solid tumors are responsible for around 90% of human cancers (Li et al., 2020; Oh et al., 2020). In 2018 alone, the four leading cancers responsible for deaths include lung, liver, colorectal, and stomach (Najafi et al., 2021). Standard-of-care therapies can well control early-stage solid cancers of non-lymphoreticular origins. Recurrent, resistant, or metastatic tumors are most commonly surgically unresectable and are usually nonresponsive to chemotherapies or radiation (Fousek and Ahmed, 2015). In recent times, alternative approaches including engineered immune cell-based therapies have shown promise in solid tumor treatment.

In solid malignancies, there is a growing interest in immunotherapies owing to the peculiar interaction between tumor complex and the immune system (Slaney et al., 2014). Indeed, the immune system plays a dual role by mediating antitumor properties through CD4^+^ and CD8^+^ T cells and their immune-activating cytokines, wherein conversely protecting the tumors from death via activating T regulatory cells as well as their immunosuppressive cytokines. Immunotherapies have greatly advanced in recent times in terms of cancer treatment via modifying the immune system to improve its capacity to detect and eradicate neoplastic cells (Papaioannou et al., 2016; Maalej et al., 2023). Adoptive cellular therapy (ACT) has a great potential and therapeutic promise in the treatment of various cancers (Grimes et al., 2021). So far, ACT has been mostly performed by utilizing 3 major cellular immunotherapies including genetically engineered tumor infiltrating lymphocytes (TILs), T-cell receptors (TCRs) T cells, and chimeric antigen receptor (CAR)-T cells (Tan et al., 2021).

ACT involves the derivation of mononuclear cells directly or peripherally from a patient’s tumor samples to expand and/or genetically modify the lymphocytes to ameliorate tumor-fighting abilities before returning the cells to the patient. So far, ACT has been performed mainly via utilizing three strategies including CAR-T cells, TCRs, and TILs (Grimes et al., 2021). FDA has approved the first TIL therapy lifileucel (Amtagvi) on February 2024 to treat advanced melanoma. Afamitresgene autoleucel (Tecelra) was the first engineered T cell therapy to gain US Food and Drug Administration (FDA) accelerated approval on August 2024 to treat patients with solid tumor. These approved therapies have the ability to harness the TCR on lymphocytes to detect and destroy cancer cells. However, there are multiple challenges involved in the usage of T cell therapies in solid tumor treatment (Fousek and Ahmed, 2015). Biotechnology industries are looking toward various approaches to overcome these challenges.

Radiotherapy is a well-established cancer treatment, which has the capacity to modulate the tumor microenvironment (TME) and mediate immune cell infiltrations (He et al., 2023). In addition, radiotherapy can trigger the release of various chemokines, improve the recognition as well as activation of NK cells, and increase the expression of various tumor-specific surface antigens (He et al., 2023). The combination of CAR T cell therapy and radiotherapy is emerging as a potential approach to improve cancer control and enhance patient outcomes (Zhong et al., 2023). It has been observed that radiotherapy can foster a TME favorable to CAR T cell infiltration. Moreover, radiotherapy can regulate this TME by decreasing the number of immunosuppressive cells (for example- M2 macrophages and regulatory T cells), and elevating the level of pro-inflammatory signals, therefore improving CAR T cell functions and infiltration (Chang et al., 2024). On the other hand, small molecule-based advanced cancer immunotherapies have been advanced in recent years. These small molecules have the ability to target specific molecular cascades within immune cells and make it easier to target the specific components of TME, which can decrease systemic toxicities and off-target effects (Bedard et al., 2020). Furthermore, the combination of immunotherapy and small molecule modulators can synergistically improve the suppressive effect of tumor progression by empowering the immune system to precisely modify responses within the TME, boosting its ability to detect and eradicate cancer cells (Singh et al., 2023).

This review article aims at useful and latest reports regarding potential engineered immune cell therapies that can be beneficial in the treatment of solid tumors, their clinical outcomes, and current challenges that need to be addressed to optimize their safety and efficacy.

## 2 Conventional therapies vs. immunotherapies in the treatment of solid tumors

Various treatment options are provided to cancer patients following their diagnosis. Several factors need to be considered while developing a suitable management plan including the patient’s physiological status, sites of cancer, and cancer type. Typical cancer treatments include radiation, chemotherapy, surgery, or a combinatorial approach. In selected scenarios, surgical resection is potentially curative, however patients with most advanced solid tumors are not suitable candidates for this approach. Multidisciplinary approaches such as radiation and chemotherapy are needed for most patients with advanced solid tumors (Guha et al., 2022). Cytotoxic chemotherapy drugs have a major limitation of causing serious side effects because of the lack of specificity, thus they attack both tumor and normal cells. On the other hand, radiation therapy is commonly utilized as part of a combination with surgery or chemotherapy, as radiation therapy alone cannot cure most cancer types (Baskar et al., 2012). Common adverse effects of radiation therapy include fatigue, stiffness, skin swelling, itchy skin, and dry skin (Cheng et al., 2019). Increasingly, cancer immunotherapies are being integrated into multidisciplinary cancer care because of their capacity to mediate promising and durable disease management. In order to include more types of solid tumors in the immunotherapy treatment regimen, more studies and advances are needed to overcome critical challenges associated with targeted delivery and immunosuppression (Guha et al., 2022).

The immune system has a significant contribution in tumorigenesis, thus the contribution of immunotherapy in the treatment of different tumor types has gained a lot of attention. Several cancer immunotherapies have already been approved in the 21st century to treat different cancer types (Guha et al., 2022). ACT has been used for a long time in the treatment of cancer and various other diseases. Indeed, the adoptive transfer of *ex vivo* expanded T lymphocytes has exhibited limited antitumor effectiveness, since these T lymphocytes have a deficiency of specificity against tumor cells (Restifo et al., 2012). In order to improve the effectiveness of ACT, the infusion of TILs with specificity against the tumor cells in individuals with preconditioning regimens markedly ameliorated the therapeutic effectiveness (Li et al., 2019). Following the cloning of the TIL’s TCR gene, now it is possible to endow T cells with definite selectivity through the transfer of cloned TCR gene (Shafer et al., 2022). In cancer treatment, engineering of T cells engineered via using viral vectors to express the TCR gene with defined selectivity has shown a substantial benefit (Kalos and June 2013).

## 3 Potential engineered immune cell therapies in the treatment of solid tumors

### 3.1 T cell receptor therapy (TCR-T)

TCR-T uses autologous T cells derived from peripheral blood mononuclear cells via leukapheresis, which is followed by TCR gene transduction (typically by using lentivirus or various other gene delivery approaches) as well as T-cell expansion. TCR-T doses are typically transfused back to cancer patients following lymphodepleting chemotherapy with cyclophosphamide and fludarabine (to mediate the delivery of cytotoxic T-lymphocytes) followed by administration of interleukin (IL)-2 (Tsimberidou et al., 2021). TCR-T has already proved its durability, effectiveness, and safety in various solid tumors such as synovial sarcoma, melanoma, and human papillomavirus-associated tumors (Ma et al., 2024). Varying success rates were obtained in a number of TCR-based trials. A objective response rate (ORR) of 61% was obtained among individuals with soft tissue sarcomas, particularly the individuals with with resistant synovial sarcomas expressing New York esophageal squamous cell carcinoma-1 (NY-ESO1) (Robbins et al., 2015). On the other hand, an ORR of 20%–60% was observed in the case of melanomas, while an ORR of 17%–64%) was observed in the case of hepatocellular carcinoma as per the patient status as well as target (hepatitis B virus [HBV] or alpha-fetoprotein antigen-targeted) (Ma et al., 2024). An enhanced disease control rate (DCR) of around 80% was observed in the trials that primarily targeted esophageal cancers, non-small-cell lung cancer, and head and neck squamous cell carcinoma (Ma et al., 2024). In addition, TCR-T exhibited its durability and effectiveness in several solid tumor niches.

Conventional first-line anthracycline-based chemotherapies showed a 3-year survival of less than 20% and only 26% ORR in the case of soft tissue sarcoma, whereas a specific antigen-based TCR-T performed better in heavily treated conditions (Judson et al., 2014). In the case of metastatic synovial sarcoma, an ORR of 35.7%–66.7% was observed with NY-ESO1-specific TCR-T, along with 5- and 3-year survival rates of 14% and 38%, respectively. In addition, this NY-ESO1-specific TCR-T was found to perform better as compared to the programmed death-1 (PD-1) inhibitor, which had an ORR of 10% only (Robbins et al., 2015; Tawbi et al., 2017; Hong et al., 2020). Metastatic human papillomavirus (HPV)-related cancers are typically standard therapy-resistant and incurable, a DCR of 83.3% and ORR of 50% were observed with the HPV E7-targeted TCR-T, which further extends the applications of TCR-T for carcinomas induced by viruses (Nagarsheth et al., 2021). Even in tumors like refractory malignant pleural mesothelioma that are targetable by TCR-T and CAR, gavocabtagene autoleucel (a novel cell therapy based on autologous, genetically engineered T cells) showed a DCR of 100% and ORR of 50% in interim analysis, in comparison with the results of a phase I clinical trial of mesothelin-targeted CARs and PD-1 antibody (a DCR of 68.8%) (Adusumilli et al., 2021).

Advantages in terms of efficacy and safety were also observed with TCR-based bispecific protein as compared to standard therapy (Nathan et al., 2021; Olivier and Prasad, 2022). In the case of hepatocarcinoma, a median overall survival of 33.5 months was observed with the HBV antigen-targeted TCR-T, where a median overall survival of 10.7 months was observed with sorafenib and a median progression-free survival of only 5 months was observed with CD133 CAR-T (Wang et al., 2018). A specific TCR-T therapy’s The safety profile mainly relies on its on-target, off-tumor (OTOT) activity or off-target toxicity. These unwanted toxicities were carefully circumvented via preclinical investigations and optimizing target selection in the most recent clinical trials. Side effects commonly associated with ACT include cytokine release syndrome (CRS) and immune effector cell-associated neurotoxicity syndrome, which were found to be milder in association with the TCR-T as compared to CAR-T. In general, TCR-T-associated side effects were found to be better tolerated because of recent developments, thus a higher tolerable dosage can be administered to ameliorate effectiveness (Ma et al., 2024). As compared to ACTs, the benefits of using TCR-T *in vivo* have been validated via exploring clinical and pre-clinical data in terms of the mechanism of action. When aimed at the same target, synthetic TCRs and antigen receptors showed earlier and improved tumor infiltration than CAR-T, which was found to be linked with enhanced antitumor effectiveness at the preclinical level (Liu et al., 2021). However, there is a lack of clinical data in terms of direct comparisons of TCR-Ts with ACTs (Ma et al., 2024).

### 3.2 Induced pluripotent stem cell (iPSC) therapies

Indeed, iPSCs have been identified as a promising source of engineered off-the-shelf allogeneic cell therapies because of their ability for clonal selection following genetic modification, comparatively easier genetic engineering, unlimited expansion capacities, and removal of the necessity to collect cells from a donor at any given time (Zhu et al., 2018; Yamanaka, 2020; Zhou et al., 2022). Over the past decade, iPSCs technology has progressed substantially and demonstrated its application in malignant solid tumors. It has been observed that iPSCs obtained from readily available cells have the ability to expand indefinitely and can also differentiate into all specialised cell types, which can provide an unlimited and strong source for the generation of differentiated cells. Moreover, iPSCs obtained from individuals with an inherited predisposition towards cancer development might mimic the early stage of tumor development and can facilitate the understanding of tumor progression (He et al., 2023).

There is a growing interest in cancer cells reprogramming into iPSCs for resetting the identification of malignant cells without modifying the cell genome sequence. Various studies have already induced the transformation of malignant solid tumor cells, such as low-grade gliomas (Liu et al., 2019), sarcoma (Zhang et al., 2013), prostate cancer (Zhang et al., 2020b), lung cancer (Mahalingam et al., 2012), and human germ cell tumors (Taguchi et al., 2021), into a pluripotent state via utilizing targeted transcription factors. This iPSC technology has confirmed the capacity to markedly decrease the tumorigenicity of the original parental cancer cells (He et al., 2023). It has also been revealed that solid tumor cells are flexible, thus the cells can be reprogrammed by utilizing iPSCs technology to reverse the malignant tumor phenotypes. Furthermore, this technology has motivated novel approaches in the treatment of malignant tumor (He et al., 2023).

### 3.3 Chimeric antigen receptor (CAR)-T cell therapies

**FIGURE 1:**
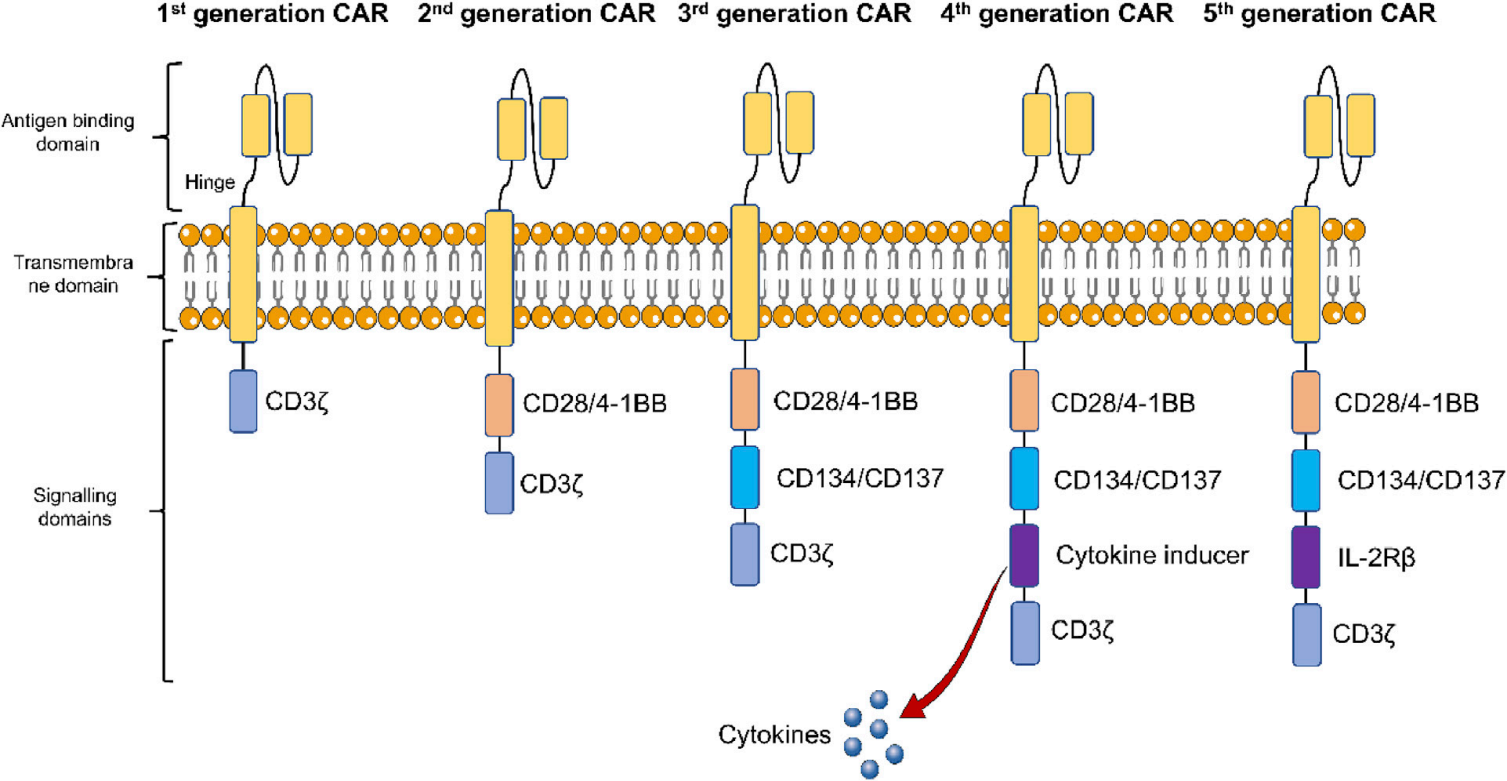
Structural components of five generations of chimeric antigen receptor (CAR)-T cells. The first-generation CAR-T cells only contain CD3ζ, an intracellular domain, while they lack co-stimulatory signals. Second-generation CAR-T cells, contain a co-stimulatory domain like CD28 or 4-1BB, while 2 or more co-stimulatory domains are involved in third-generation CAR-T cells. The fourth-generation CAR-T cells include expressions of certain cytokines. Co-stimulatory domains activating various other signalling cascades are incorporated in the fifth-generation CAR-T cells.

In the past few decades, CAR-T cell-based therapies have revolutionized cancer therapy, since they are capable of producing durable and effective clinical responses (June et al., 2018). It is now well-known that CARs are engineered synthetic receptors that can redirect T cells to detect and eradicate the cells that express targeted antigens (Sterner and Sterner, 2021). There are 3 major functional domains present in the CAR structure including the intracellular domain, transmembrane domain, and extracellular domain (Figure 1). An intracellular domain containing only CD3ζ is present in first-generation CARs, while they lack co-stimulatory signals (Lindner et al., 2020). In contrast, a co-stimulatory domain like CD28 or 4-1BB is present in second-generation CARs, while 2 or more co-stimulatory domains are involved in third-generation CARs. On the other hand, the fourth-generation CARs were developed as per the second-generation CAR, which includes expressions of certain cytokines. Finally, co-stimulatory domains activating various other signalling cascades are incorporated in the fifth-generation CARs (Chen et al., 2024).

**FIGURE 2:**
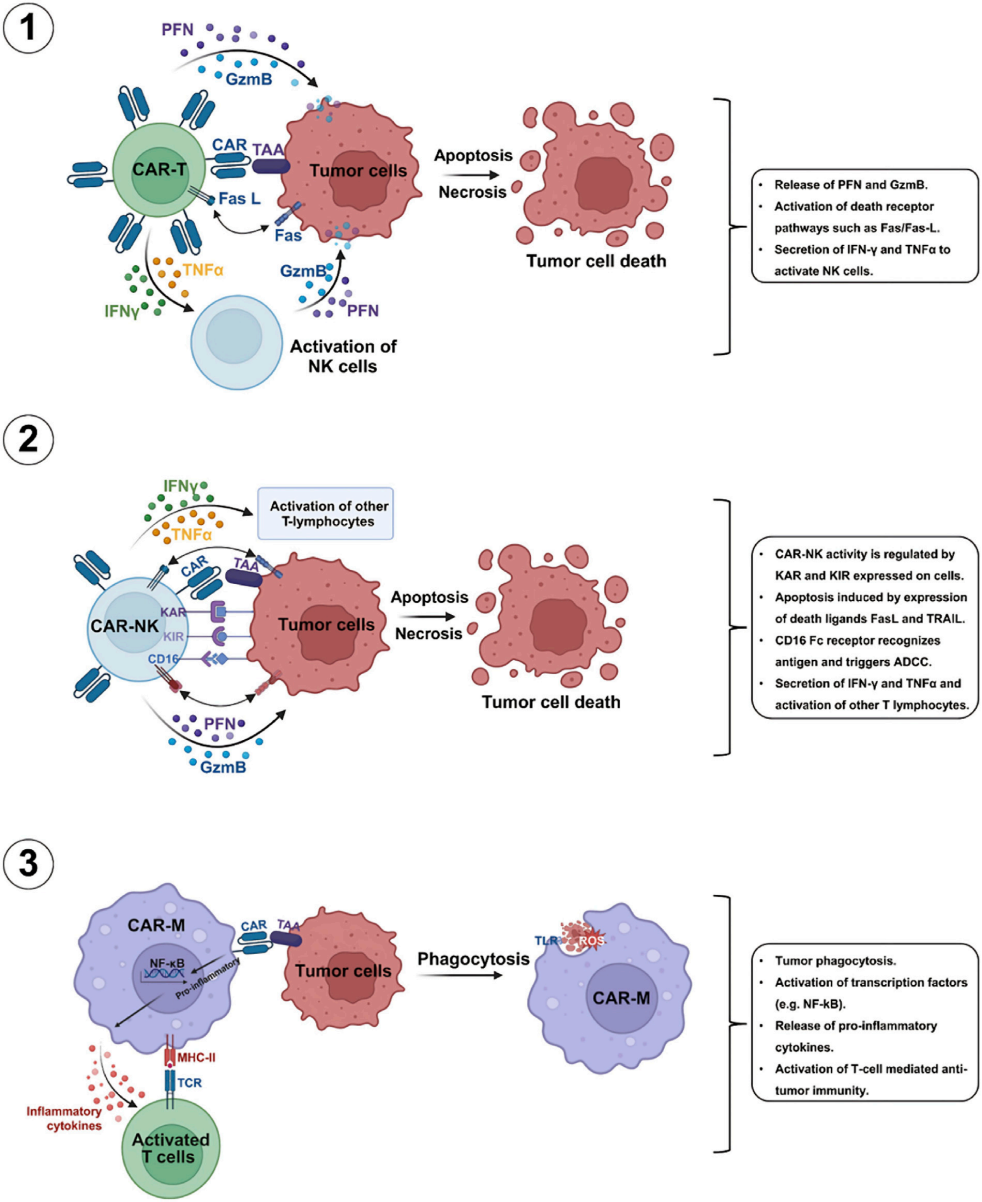
Mechanisms of commonly used CAR-T cells mediated killing processes of tumor cells. Various commonly used CAR-T cells including CAR-T, CAR-NK, and CAR-macrophage (CAR-M) possess the capacity exhibit strong antitumor properties through several mechanisms [Abbreviations: GzmB, granzyme; -γ, interferon-gamma; TNF-related apoptosis-inducing ligand; ADCC, antibody-dependent cellular cytotoxicity; IFNKAR, killer activation receptor; KIR, killer inhibitory receptors; PFN, perforin; TRAIL-R, TNFα, tumor necrosis factor-alpha]. Reproduced with permission from Elsevier, (Zhang et al., 2024).

The use of CAR-T is well-established in cancer treatment, thus the use of CAR engineering to alter other types of immune cells has greatly motivated researchers (Figure 2). In the case of solid tumors, most of the earlier phase clinical trials utilized second-generation CAR T-cell-based therapies, however limited antitumor properties were observed as compared to what was observed in blood cancers (Srour and Akin, 2023). Therefore, two costimulatory domains were incorporated in third-generation CARs to enhance the antitumor properties (Sadelain et al., 2013). Remarkable outcomes obtained with CAR T-cell-based therapies in blood cancers encouraged an expectation for similar outcomes in the case of solid tumors. A growing number of preclinical and clinical studies over the past few years have explored the mechanisms and applications of CAR T-cells in the case of solid tumors (Dhaliwal et al., 2024). Even though their effectiveness in solid tumor treatment is yet to be demonstrated, numerous tumor-linked neoantigens and antigens have been detected as potential targets (Keshavarz et al., 2022).

### 3.4 Tumor infiltrating lymphocyte (TIL) therapy

TIL therapy is an outstanding immunotherapeutic approach, which provides prospects for the management of difficult cancers (Hong et al., 2024). TILs are mononuclear cells that occur naturally and infiltrate the solid TME, which play roles as part of the broader group of immune cells at the sites of tumors (Savas et al., 2016; Mohammad Hossein et al., 2022). In the case of TIL therapy, lymphocytes are extracted from a tumor and then expanded outside of the body (*ex vivo*), which are then reintroduced to improve immune responses against tumor cells (Betof Warner et al., 2024). TILs efficiently eradicate cancer cells and have less chance to cause injury to normal cells, offering greater therapeutic potential with fewer side effects, therefore they have superior therapeutic properties along with lesser side effects (Hong et al., 2024). In humans, the first use of TIL therapy resulted in a 60% regression in the case of metastatic melanoma (Dhaliwal et al., 2024).

Solid tumors were found to be highly heterogeneous and they often do not contain an ideal tumor marker, notwithstanding blood cancers along with lineage-specific markers (Roshandel et al., 2021; Wang et al., 2021). Interestingly, TILs are polyclonal cells containing various receptors thus able to detect multiple tumor-associated antigens, therefore TILs as genetically-modified immune cells show superiority in the treatment of solid tumors. Immune escape and heterogeneity of tumors can be overcome by TILs, which can offer better clinical responses as compared to CAR-T cell-based therapies in the treatment of solid tumors with greater mutation rates, for example, melanoma (Titov et al., 2021). In addition, within the TME, TILs show greater tumor-specificity and have the capacity to target even unknown tumor neoantigens, which removes the need for previous understanding regarding major histocompatibility complex (MHC) restriction or tumor-associated antigens (Fernandez-Poma et al., 2017).

Stromal TILs (sTILs) and intratumoral TILs (iTILs) are the major types of TILs. It has been observed that sTILs are easily detectable and commonly found in the tumor stroma, while iTILs are rarely found in tumor cell clusters thus their identification process is complex (Savas et al., 2016). Most of the TILs are effector memory T cells that show high effectiveness in antitumor properties and proliferation, which are activated by tumor-associated antigens *in vivo* and can also proliferate *in vitro* up to 10^5^ times. Since TILs have the capacity to infiltrate TME, thus they contain chemokine receptors that are required for migration toward the TME following administration (Fernandez-Poma et al., 2017). Lower off-target toxicity is another advantage provided by TILs as compared to CAR-T cells, which perhaps owing to the negative selection of TCRs during T cell maturation (Wang et al., 2021).

### 3.5 Mesenchymal stem cells (MSCs)

MSCs are self-renewing, versatile cells that can be obtained from various sources, for example, bone marrow, amniotic fluid, adipose tissue, and umbilical cord (Zhang et al., 2020a). MSCs has shown promising outcomes in cancer immunotherapy via providing oncolytic immunotherapy and increasing CAR-T cell activities, thus being able to exert substantial antitumor actions. Exosomes derived from MSCs might possess similar properties (Hombach et al., 2020). Nonetheless, varying research outcomes have been observed regarding the capacity of MSCs to modify CAR-associated products. Perplexingly, the role of MSCs has also been indicated in mediating metastasis and tumor growth in certain scenarios (Holthof et al., 2021). MSCs are currently being investigated as a delivery vehicle for various therapies including oncolytic viruses (Zhu et al., 2017) and tumor necrosis factor (TNF)-related apoptosis-inducing ligands (Shaik Fakiruddin et al., 2018).

Former studies involving tumors and MSCs were mainly associated with the exploration of the effects of naive (unmodified) MSCs (N-MSCs) on tumors. It has been observed that N-MSCs can be isolated from various natural tissue sources and can be homed to tumors to efficiently target the TME and assess their uses as antitumor agents. In addition to this, N-MSCs can be co-cultured with *in vitro* tumor cells, which may suppress the proliferation of tumor cells (Shams et al., 2023). In a study, it was confirmed that N-MSCs may avert *in vitro* proliferation of solid tumors and leukemia cell lines (Ramasamy et al., 2007). Furthermore, the suppressive effect of N-MSCs was found to be dose-dependent, and the suppression rate was decreased at higher proportions of N-MSCs (Ramasamy et al., 2007). Future studies should optimize their engineering, clarify the contribution of MSCs in tumor growth, and explore them as part of combination therapies (D’Avanzo et al., 2024).

### 3.6 Natural killer (NK) cell therapy

**FIGURE 3:**
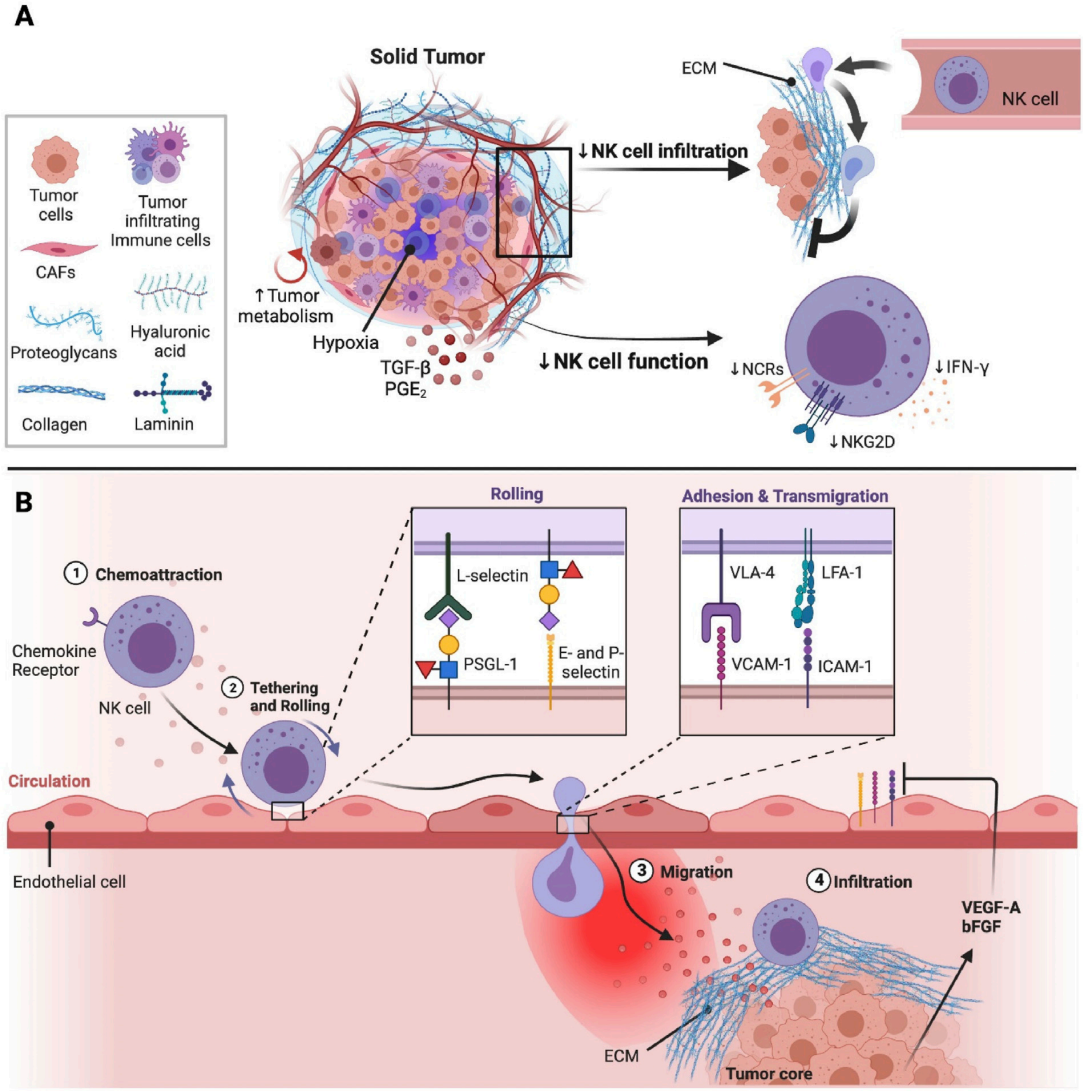
The effect of tumor microenvironment (TME) on natural killer (NK) cell infiltration and activities at the solid tumor sites (Portillo et al., 2023) **(A)** Solid tumors are usually composed of immunosuppressive immune cells, tumor cells, and cancer-associated fibroblasts (CAFs), which can suppress the activities and infiltration of NK cells. CAFs mainly generate the components needed for the extracellular matrix (ECM). Different components such as laminin, hyaluronic acid, proteoglycans, and collagen are present in the ECM, which play a role in solid stress and tumor stiffness. Tumor vasculature can be obstructed by solid stress, which can result in a hypoxic condition in the TME. In addition, the high energy necessities of rapidly proliferating tumor cells can result in a poor supply of nutrients in the TME, which can further reduce the metabolic fitness and anti-tumor properties of NK cells. Immunosuppressive elements including prostaglandin E2 (PGE2) and transforming growth factor beta (TGF-β) are also present in TME, which can further suppress the functions of NK cells via reducing the signalling and expression of activation receptors. **(B)** Hypoxia in the TME can result in the generation of various pro-angiogenic factors such as vascular endothelial growth factor (VEGF)-A and basic fibroblast growth factor (bFGF), which can decrease the expressions of adhesion molecules on endothelial cells via weakening extravasation of NK cells into the sites of tumors. Following the migration of NK cells towards tumors, NK cells are shielded from tumor cells through the stiff and dense ECM surrounding the tumor core (Portillo et al., 2023). Integrins including very late activation antigen-4 (VLA-4) and Lymphocyte function-associated antigen 1 (LFA-1) are found to be expressed on the surface of NK cells that bind with intercellular adhesion molecule 1 (ICAM-1) and vascular cell adhesion molecule-1 (VCAM-1) to mediate strong binding with the endothelium, followed by extravasation into the tissues (Harjunpää et al., 2019).

Unlike T cells, NK cells have the capacity to detect and target various abnormal or stressed cells without preceding sensitization, such as metastatic and MHC-I-deficient tumor cells (Laskowski et al., 2022). In recent times, NK cell engagers have markedly advanced NK cell therapy, which can direct NK cells precisely to tumors (Vivier et al., 2024). Methods on *ex vivo* cytokine induction are also utilized to increase NK cell activities and offer a memory-like phenotype, such as feeder cell approaches by utilizing soluble IL-12, -15, and −18 and membrane-bound IL-15 (Terrén et al., 2022). In the case of solid tumors, there is a high chance of the occurrence of abnormal tumor vasculature, where solid stress caused by the growing tumor may compress tumor vasculature to reduce blood flow into the tumor bed (Figure 3) (Portillo et al., 2023). Another drawback of using NK cell-based therapies is the shorter duration, which can decrease their long-term therapeutic effectiveness and might necessitate repeated administrations (Terrén et al., 2022).

Furthermore, the therapeutic effectiveness of NK cells is largely determined by their sources, where functional heterogeneity can influence clinical outcomes. In recent times, NK cells derived from cord blood have indicated this issue, which confirmed that a higher level of effector-related genes is present in NK cells derived from optimal cord blood units (CBUs) and showed enhanced activities than NK cells derived from suboptimal CBUs (Marin et al., 2024). Still, there is a debate regarding the requirement for conditioning regimens in the case of allogeneic NK cell therapies, therefore more clinical trials are needed to elucidate their requirement (Kerbauy et al., 2021). More studies are also essential to improve the trafficking of NK cells, effector capacity, and metabolic profiles (Laskowski et al., 2022; Berrien-Elliott et al., 2023; Marin et al., 2024). Other important areas that need to be considered include maintaining cell viability after cryopreservation and the development of a scalable manufacturing process. Along with CAR-NK cell therapy, novel approaches are also emerging, for example, the combination of a bispecific CD30/CD16 antibody along with blood-derived NK cells and cytokine-activated cord blood (Kerbauy et al., 2021).

Cytokine-induced killer (CIK) cells also have already demonstrated their effectiveness and an outstanding safety profile in several clinical trials, even across HLA barriers in an allogeneic setting (Magnani et al., 2020; Wu and Schmidt-Wolf, 2022). CIK cells showed strong anti-tumor ability against several solid and hematological malignancies (Sharma et al., 2024). In addition to this, CIK cells show a heterogenous T cell population with a mixed NK cell phenotype and combine adaptive T cell-mediated with MHC-unrestricted functions of the innate immune system (Moser et al., 2025). CIK cells were found to be compatible with nearly all kinds of immune checkpoint inhibitors, epigenetic drugs, and CAR-CIK therapy. It has been reported that CAR-CIK therapy is at least as effective as CAR-T cells. In addition, CAR-CIK therapy has favorable allogeneic applicability and a safety profile (Moser et al., 2025).

### 3.7 Dendritic cell (DC) therapies

Various endogenous danger signals can trigger an immune response, including fragments of dying cells and microbial products (known as pathogen-associated molecular patterns). These danger signals are detected by various immune cells (Li and Wu, 2021). Among them, DCs play a role as the major link between adaptive and innate immune responses. It has been observed that pulsing DCs with whole tumor cell lysates *in vitro* and *in vivo* can trigger therapeutic antitumor immune responses following vaccination (Rosenblatt et al., 2011). Nonetheless, there are several challenges (such as antigen loading and method optimization for DC generation) that need to be addressed before using DC-based therapies to treat solid tumors (Jung et al., 2018; Sheykhhasan et al., 2025). Interestingly, blocking suppressive molecules like PD-1, cytotoxic T-lymphocyte-associated protein 4 (CTLA-4), and programmed death-ligand 1 (PD-L1) on immune and tumor cells restored tumor-specific T cell functions (Baumeister et al., 2016; Vreeland et al., 2016). In a clinical trial, administration of a combination of radiotherapy (35 Gy) and *in situ* DC therapy utilizing GM-CSF in individuals with metastatic solid tumors triggered an abscopal effect in 11 of 41 participants, which was markedly greater than the abscopal effects mediated by radiotherapy alone (Golden et al., 2015).

There are several approved checkpoint inhibitors that are used in solid tumor treatments owing to their outstanding clinical outcomes such as anti-PD-L1 (avelumab, durvalumab, and atezolizumab), anti-CTLA-4 (durvalumab and ipilimumab), and anti-PD-1 (nivolumab and pembrolizumab), It has been reported that effectiveness of these checkpoint inhibitors, particularly the monoclonal antibodies that block PD-L1, usually linked with the mutational burden, expression of PD-L1 in the TME, and the number of TILs (Snyder et al., 2014; Tumeh et al., 2014; Dammeijer et al., 2017; Fennell et al., 2018; Yi et al., 2018). DC-based therapies enhance the infiltration of CD8^+^ T cells that are specific to tumors and increase the expressions of PD-1 on these TILs, which may make tumors with lower numbers of TILs receptive to anti-PD-L1 therapy (Antonios et al., 2016). Most of the DC-based clinical trials were successful in producing tumor-specific CTLs in individuals with cancers, however the activities against most solid tumors were found to be rather disappointing (Butterfield, 2013). There are several factors that can result in insufficient efficacy of DC vaccine-mediated immune responses against solid tumors. One such factor is the inadequate number of CD8^+^ CTL induction in response to DC vaccination alone (Lou et al., 2004). CTLs produced in this manner might contain suboptimal antitumor properties *in vivo*, perhaps because of inadequate migration or weak activation at the tumor sites. The responsiveness of such cells to host-derived regulatory processes also seems to be an issue (Jung et al., 2018).

### 3.8 Chimeric antigen receptor (CAR) macrophages (CAR-Ms)

Multiple limitations of CAR-T cell-based therapies include graft *versus* host disease, OTOT toxicity, immune effector cell-associated neurotoxicity syndrome, CRS, time-consuming production, and high cost (Bonifant et al., 2016; Yadav et al., 2020). In solid tumor treatment, CAR-T cell has limited effectiveness because of various reasons including high complexity of TME, incompetent homing and infiltration, limited T cell fitness, antigen escaping, and heterogeneity (Wagner et al., 2020). Macrophages have the capacity to exert various effector activities that can mediate support tumor clearance. In recent times, CAR-Ms have been generated by the genetic engineering of macrophages to express targeted proinflammatory transgenes (Brempelis et al., 2020; Gardell et al., 2020; Kaczanowska et al., 2021). CAR-M has emerged as a potential therapy and its use can prove beneficial in the solid tumor treatment (Hadiloo et al., 2023). In the case of both *in vitro* and *in vivo* studies, CAR-Ms have resulted in great results in the of solid and blood cancer treatment. Indeed, CAR-Ms were found to possess strong anti-cancer properties as compared to macrophages alone or various other macrophage-based therapies (Liu et al., 2022). Several studies demonstrated significant outcomes of cytotoxicity in the CAR-manner through multiple target antigens including CD19 (Morrissey et al., 2018), mesothelin (Anderson et al., 2022), human epidermal growth factor receptor 2 (HER2) (Klichinsky et al., 2020), transmembrane glycoprotein mucin 1 (MUC1) (Eisenberg et al., 2021), and GD2 (Zhang et al., 2023).

**FIGURE 4:**
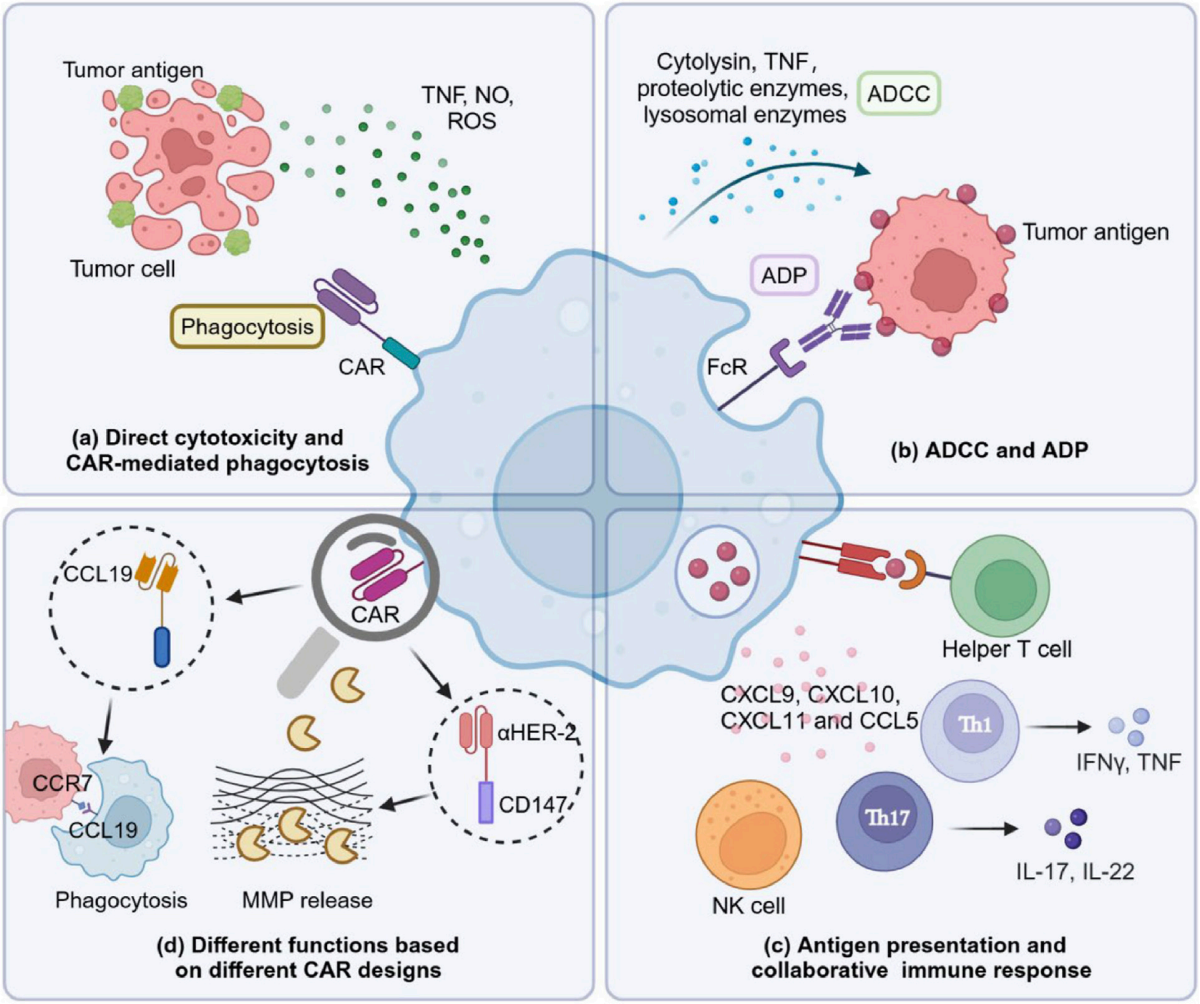
Killing mechanisms of chimeric antigen receptor (CAR) macrophages (CAR-Ms) (Lu et al., 2024). **(a)** CAR-Ms show direct cytotoxic properties via secreting releasing nitric oxide (NO), reactive oxygen species (ROS), and tumor necrosis factor (TNF). **(b)** CAR-Ms can cause direct cytolysis of tumor cells via antibody dependent cellular cytotoxicity (ADCC) and antibody dependent phagocytosis (ADP) by binding with Fc receptor (FcR) expressed on its surface to antibodies coated on the tumor cell membranes. **(c)** Extensive communication between CAR-Ms and other immune components including helper T cells have a significant contribution in tumor clearance. CAR-Ms can play a role as an antigen-presenting cell by presenting tumor antigens to prime T cells. In addition, CAR-Ms can enhance intratumoral infiltration of various tumor infiltrating cells including antitumoral neutrophils, T helper 17 (Th17) cells, natural killer (NK) cells, T helper 1 (Th1) cells, and cytotoxic T cells (CTL) via releasing C-C motif chemokine ligand 5 (CCL5), and C-X-C motif chemokine ligand (CXCL)-8, -9, -10, and -11. **(d)** Macrophages can exert different roles depending on the CAR design. CCL19-CAR-Ms can mediate the engulfment of C-C chemokine receptor type 7 (CCR7)-positive tumor cells to slow down tumor progression as well as metastasis. Human epidermal growth factor receptor 2 (HER2) CAR-Ms modified with a cluster of differentiation 147 (CD147) can induce matrix metalloproteinase (MMP) release to damage the extracellular matrix, which can ultimately lead to the infiltration of more immune cells (Lu et al., 2024).

CAR-Ms and their killing capacity can regulate and modify the immune system and associated factors to enhance their anti-cancer properties. CAR-Ms can directly cause cytotoxicity in tumor cells (Figure 4). Macrophages activated by lipopolysaccharide were found to release various harmful substances that can cause the disintegration of tumor cells, such as nitric oxide, reactive oxygen species, and TNF (Lu et al., 2024). A number of preclinical studies of CAR M cells confirmed substantial anti-tumor properties in the case of both *in vitro* and *in vivo* studies. For instance, CAR M exerted anti-tumor properties on leukemia cells through luciferase reporter assays or ovarian cancer cell line HO8910 expressing a high level of mesothelin *in vivo* (Zhang et al., 2020a). On the other hand, MUC1-targeting CAR-Ms exhibited strong anti-tumor activities via phagocytosis and release of various pro-inflammatory cytokines including TNFα, IL-8, and IL-1β in the presence of MUC1 expressing tumor cells from malignant pleural effusions or solid lung tumors (Eisenberg et al., 2021). Despite their therapeutic promises, there are several limitations of CAR-Ms in terms of solid tumor treatment because of the complex TME and unique features of solid tumors. Even though CAR-Ms showed good outcomes in several preclinical studies, multiple problems were faced afterwards including cell exhaustion, the suppressive activities of TME, antigen escape, and tumor heterogeneity. Nonetheless, human cancer-associated TMEs possess a more complex scenario as compared to animal models (Kochneva et al., 2020; Toor et al., 2020).

## 4 Clinical trials of engineered immune cell-based therapies in the treatment of solid tumors

Cellular immunotherapies involve several approaches for the *ex vivo* manipulation of immune cells, such as TILs, TCR-T cells, CAR-T cells, iPSCs, MSCs, NK cells, DCs, and CAR-Ms (Albarrán-Fernández et al., 2025). Delivery of unselected TILs have the capacity to exert clinically significant responses in advanced melanoma (Table 1), even in individuals refractory to anti-PD-1 antibodies (Martín-Lluesma et al., 2024). In addition, lifileucel is an FDA-approved TIL therapy for the treatment of solid tumors. TIL-based therapies also exhibited promising outcomes against various other malignancies, such as cervical cancer (Stevanović et al., 2019) and non-small cell lung cancer (Creelan et al., 2021). Candidates for TIL-based therapies go through resection of tumor lesions, from which T cells are derived, expanded as well as reinfused after lymphodepletion, typically followed by administration of systemic IL-2 to mediate *in vivo* survival and expansion of T cells (Yossef et al., 2023). A number of clinical trials are currently ongoing that are exploring the potential of a range of engineered immune cell therapies in the treatment of solid tumors. Some of the clinical trials are already showing promising results, however there is a general lack of substantial clinically significant responses and occurrence of OTOT toxicity. A summary of currently ongoing clinical trials evaluating engineered immune cell therapies has been provided in Table 2.

**TABLE 1:** Selected successful clinical trials with engineered immune cell therapies in the treatment of solid tumors.

Therapy type	Indications	Study type	Number of study participants	Study outcome	FDA approval status	Clinical trial number
TCR-engineered T (TCR-T) cell therapy (Afamitresgene autoleucel)	Ovarian or urothelial, melanoma, non-small cell lung, head and neck, gastric, esophagogastric junction (EGJ), esophageal, and endometrial cancers	Ongoing Phase 1 trial	120	Exhibited acceptable safety profile; antitumor activities have been observed in individuals with head and neck cancer and EGJ cancer	FDA-approved for unresectable or metastatic synovial sarcoma	NCT04044859
Tumor infiltrating lymphocytes (TILs) (lifileucel)	Advanced melanoma	Multicohort, prospective, phase II, multicenter study	153	TILs exhibited a clinically significant role in heavily pre-treated individuals with advanced melanoma with an advanced disease and high tumor burden	FDA approved for melanoma	NCT02360579
Autologous dendritic cell vaccine (sipuleucel-T)	Metastatic, asymptomatic, hormone-refractory prostate cancer	Double-blind, randomized, placebo-controlled, phase III trial	98	Patients receiving the sipuleucel-T were found to have three times more activated T cells in prostatectomy specimens than the control group	FDA approved for metastatic castrate-resistant prostate cancer	NCT01133704

**TABLE 2:** A summary of selected ongoing clinical trials with engineered immune cell therapies in the treatment of solid tumors.

Therapy type	Indications	Study type	Estimated participants	Estimated completion	Clinical trial number
Chimeric antigen receptor (CAR)-T cell therapy	Paediatric patients with high risk and/or relapsed and/or relapsed/refractory neuroblastoma	Phase I and phase II trials	42	February 2027	NCT03373097
Dendritic cells vaccine and atezolizumab	Epithelioid malignant pleural mesothelioma	Single arm phase I/II trial	15	October 2026	NCT05765084
Autologous tumor infiltrating lymphocytes	Solid tumors	Multi-cohort, multicenter prospective, non-randomized, open-label, Phase II study	178	August 2029	NCT03645928
EGFR806 CAR-T cell immunotherapy	Children and young patients with refractory or recurrent ​solid tumors	Non-randomized phase I, open-label, trial	44	June 2040	NCT03618381
CAR-T cell therapy	Advanced sarcoma	Interventional phase 1 trial	36	July 2032	NCT00902044
CAR-T cell therapy	Children and young patients with refractory or recurrent ​solid tumors	Open-label, non-randomized, phase I trial	68	December 2040	NCT04483778
CAR-T-EGFR-IL13Ra2	Recurrent glioblastoma (GBM)	Open-label, phase I trial	18	December 2039	NCT05168423
CAR modified T cells	Multiple Myeloma	Phase I trial	17	August 2025	NCT04555551
Glypican 3 (GPC3)-specific CAR expressed in T cells	Pediatric solid tumors	Phase I trial	10	February 2037	NCT02932956
Autologous CAR-T cell therapy	Pediatric solid tumors	Phase I trial	32	March 2027	NCT04897321
Interleukin (IL)-15 and −21 armored GPC3-specific CAR expressed in T cell therapy	Pediatric solid tumors	Phase I trial	24	July 2041	NCT04715191
P-MUC1C-ALLO1 Allogeneic CAR-T cell therapy	Metastatic or advanced solid tumors	Phase I trial	180	April 2039	NCT05239143
IL-15 and IL-21 armored GPC3-specific CAR expressed in T cell therapy	Pediatric solid tumors	Phase I trial	24	February 2040	NCT04377932
EGFR/B7H3 CAR-T therapy	Triple-negative breast cancer and lung cancer	Phase I trial	30	May 2035	NCT05341492
Claudin 6 (CLDN6) -specific CAR-T therapy	Relapsed or refractory solid tumors	Multicenter, Phase I, open-label, dose escalation trial	145	January 2040	NCT04503278

## 5 Current challenges in solid tumor treatment with engineered immune cells

Solid tumors show startling tumor-associated antigen heterogeneity and an immunosuppressive TME, which imposes a challenge for immune cells that attempt to penetrate tumors. Furthermore, solid tumors are well-supported by a complex TME capable of inhibiting immune responses and they often occur in regions within the body that are difficult to access for treatment. In order to overcome these challenges, more sophisticated engineered immune cells are required for solid tumor treatment (Fousek and Ahmed, 2015).

In general, therapies targeting a tumor profile instead of a specific tumor-associated antigen might prove more beneficial in the treatment of solid tumors. In contrast with blood tumors, solid tumors often occur in severely restricted regions within the body. For example, gliomas and various other central nervous system tumors are often challenging to treat, since systemically infused cells ought to have capacity to penetrate the blood-brain barrier to gain access to the tumor. Thus, the dose of the therapies efficiently reaching the tumor sites might be markedly decreased from the dose originally administered. Therefore, studies are increasingly focusing on the homing ability of T cells via expressing various chemokine receptors (ClinicalTrials.gov, 2012). As the engineered immune cells improve, tumors can also adapt, which can lead to immune evasion. Furthermore, tumor cells can inhibit an immune response via elevating their expressions of important anti-inflammatory signals. Therefore, extensive and continuous studies are required to overcome this tumor adaptation and to enable engineered immune cell therapies to continuously exert their antitumor properties (Fousek and Ahmed, 2015).

The use of T cells expressing transgenic T cell receptors (tgTCR) resulted in early success in the treatment of solid tumor, however clinical reports involved OTOT toxicity (Morgan et al., 2010; Linette et al., 2013). The safety profile of next-generation T cell-based therapies can be significantly improved by eliminating overly activated cells, controlling CRS, and including new systems into CAR molecules to avert OTOT toxicity (Fousek and Ahmed, 2015). More studies involving these safety measures are likely to result in widespread usage of CAR-T cell therapies in the treatment of solid tumors.

In the case of melanoma and myeloma, a high-affinity TCR targeting melanoma-associated antigen 3 showed an unwanted cross-reactivity via detecting titin, a giant muscle protein expressed in both cardiac muscles, which eventually led to cardiotoxicities and resulted in the death of two patients (Linette et al., 2013). Similar adverse effects were also observed with CAR-T cell therapy, as revealed by clinical reports (Morgan et al., 2010; Maus et al., 2013). Several doses of mesothelin-targeted CARs were developed by using an RNA-based platform to administer in an individual with pancreatic adenocarcinoma and 3 individuals with malignant pleural mesothelioma (Maus et al., 2013). Among them, a patient developed anaphylaxis to the administered cells upon infusion of the third dose. It was concluded that anaphylaxis occurred due to the generation of CAR-specific IgE antibodies, thus more studies are required with the dosing schedules that involve repeated administrations of CAR-T cells (Maus et al., 2013). In order to avoid such catastrophic events, it is important to select a suitable target tumor-associated antigen.

In addition, optimizing the specificity and affinity of the CARs or tgTCRs, preparatory regimens, and doses before the immune cell therapies (Fousek and Ahmed, 2015). CRS is a potentially life-threatening complication, which is observed with certain immunotherapies, predominantly CAR T-cell therapy. CRS can be fairly well controlled through the administration of targeted immunosuppressive agents, steroid therapy, or tocilizumab (an anti-IL-6 antibody) (Lee et al., 2014).

On the other hand, the manufacturing methods for CAR-T cells require major alterations by focusing on enhanced and streamlined production (Ramamurthy et al., 2024). At present, the manufacturing process of autologous CAR-T cell products includes genetic alteration of a patient’s T cells with viral vectors and successive *ex vivo* expansion in bioreactors with a range of cytokines including IL-2, IL-7, and IL-15, and anti-CD3/CD28 beads (Ramamurthy et al., 2024). Nonetheless, this technique involves limitations in scaling up CAR-T cell-based manufacturing because of its high cost, complexity, and customized nature (Li et al., 2020; Papathanasiou et al., 2020).

The clustered regularly interspaced short palindromic repeats (CRISPR) technology has revolutionized various fields including immunology and cancer. CRISPR-based screening and gene editing have empowered direct genomic manipulation of a range of immune cells, which has mediated unbiased functional genetic screens. Indeed, these screens have facilitated the discovery of novel factors that control and reprogram immune responses, thus providing novel drug targets (Zhou et al., 2023). On the other hand, developments in micro-/nano-technology, nanomedicine, and biomaterials have mediated the development of improved local delivery systems for cancer immunotherapy, which can further improve treatment efficacy while lessening toxicity. Moreover, locally administered cancer therapies combining immunotherapy with phototherapy, radiotherapy, or chemotherapy have the potential to attain synergistic antitumor actions (Abdou et al., 2020).

## 6 Future directions

Complex manufacturing process, higher economic costs, and need for advanced equipment are the major challenges associated with the use of engineered immune cells in the treatment of solid tumors. Centralized manufacturing at specialized institutions might help in regulatory compliance, however this process needs cryopreservation of the products for extensive inter-center coordination and transportation (Albarrán-Fernández et al., 2025). Alternatively, the point-of-care manufacturing process is can be implemented by allowing the local manufacturing of advanced cell therapies, which will significantly increase accessibility and lower production costs. Nonetheless, unlike native cells, the capacity of immune cells to be genetically engineered with various environment-responsive and controllable functions allows immune cell therapies to make alterations in the TME, which cannot be attained by conventional therapies. Moreover, extensive studies focusing on engineering immune cell therapies directly *in vivo* or using engineered off-the-shelf third-party cell sources might remove some or all of the cell therapy-associated practical challenges (Kirtane et al., 2021). A diverse scientific collaborative effort is required to unleash the full potential of engineered immune cell therapies. Remarkable advances are continuously being achieved through extensive research in molecular biology, synthetic biology, oncology, and immunology. As the outcomes of clinical trials continue to be revealed, there is a scope for using computational modelling for predictions of important parameters to be optimized in cell therapy as well as machine learning-based data analysis. The interactions of engineered immune cell therapies with the nervous system and endogenous immune system, and the impact of microbiome on the outcomes of cell therapies are the areas that might lead to novel discoveries (Kirtane et al., 2021; Schupack et al., 2022).

## 7 Conclusion

Genetic engineering empowers the enhancement of adoptively transferred cells by modifying their phenotypes and functionality through a range of mechanisms. In recent times, cell engineering approaches have advanced in modifying the TME, preventing tumor escape, enhancing tumor-targeting specificity, and improving the antitumor properties of engineered immune cells. Several engineered immune cells exhibited promising outcomes in clinical trials and numerous clinical trials are ongoing as well. However, there are several challenges in improving their efficacy in the treatment of solid tumors including identification of optimal combination approaches, optimization of the manufacturing process, development of true off-the-shelf therapies, and mitigation of side effects.
